# Psychometric properties of the COVID-19 Yorkshire Rehabilitation Scale: Post-Covid-19 syndrome in Iranian elderly population

**DOI:** 10.1186/s12879-024-08991-0

**Published:** 2024-01-11

**Authors:** Negar Tamadoni, Afsaneh Bakhtiari, Hossein-Ali Nikbakht

**Affiliations:** 1 Student Research Committee, Babol University of Medical Sciences, Babol, Iran; 2 Social Determinants of Health Research Center, Babol University of Medical Sciences, Babol, Iran

**Keywords:** Covid-19, COVID-19 syndrome post-acute, Elderly

## Abstract

**Background:**

This study aimed to assess the construct validity and reliability of the Iranian version of the COVID-19 Yorkshire Rehabilitation Scale (C19-YRS) among the elderly population.

**Method:**

A cohort of 230 elderly individuals who tested positive for Covid-19 via PCR were administered a health and demographic information questionnaire along with the C19-YRS. Both exploratory and confirmatory factor analyses were conducted, and Cronbach’s alpha was calculated.

**Results:**

Findings from the exploratory and confirmatory factor analyses of the C19-YRS revealed alterations compared to the original version, resulting in an adapted version with three factors achieved by redistributing the questions. These factors accounted for 57.46% of the total variance. Despite a relatively lower factor loading in the 6th question, it was retained due to its significance among the elderly. The Cronbach’s alpha for the C19-YRS subscales ranged from 0.730 to 0.890, indicating acceptable reliability.

**Conclusion:**

The validation results indicated a well-adjusted factor structure and internal consistency, affirming the utility of this tool among the elderly population. Consequently, the C19-YRS in Iran can serve as a valuable resource in healthcare settings, aiding in the assessment of chronic complications arising from Covid-19 in the elderly. It can be utilized as an initial screening or triage test and to evaluate the effectiveness of interventions.

**Supplementary Information:**

The online version contains supplementary material available at 10.1186/s12879-024-08991-0.

## Introduction

The world has witnessed subsequent waves of COVID-19, leading to a substantial number of patients experiencing lingering symptoms even months after recovering from the disease, commonly referred to as “Post-Covid-19 Syndrome” or “Prolonged Covid” [1]. This condition, initially termed Long COVID in 2020 by affected patient groups, describes persistent symptoms following the illness. Over time, several descriptors have emerged to define this syndrome, such as post-acute COVID-19 syndrome or post-COVID conditions [2]. In an attempt to establish uniformity in terminology, both the CDC and WHO have advocated for the broader use of “post-Covid conditions” as an umbrella term encompassing consequences persisting beyond 4 weeks post-acute infection. In contrast, the UK National Institute for Care Excellence (NICE) has introduced “prolonged COVID” as an operational definition, encapsulating ongoing symptoms persisting 4 weeks after acute infection without an alternative diagnosis. This terminology encompasses “persistent symptomatic COVID-19” for signs and symptoms between 4 and 12 weeks post-acute infection and “post-Covid syndrome (PCS)” for symptoms persisting beyond 12 weeks [3].

Prolonged Covid can affect any patient, irrespective of the initial infection’s severity, including those who were asymptomatic. However, it appears that the severity of the acute infection might elevate the risk. This condition is associated with medium- and long-term health consequences [1]. Studies conducted in the United States, based on the population, have revealed that individuals hospitalized for Covid-19 require more extensive follow-up medical care, experience higher rates of hospital readmissions, and, tragically, some have succumbed to the illness [3]. With over 674 million global cases of SARS-CoV-2 infection reported [4] and approximately 7.5 million cases in Iran alone [5], even if a conservative estimate suggests an incidence of 10–20% in developing these clinical disorders, the disease is estimated to have impacted approximately 750,000–1,500,000 people in Iran. This highlights an urgent health concern that requires immediate clinical attention.

As per the WHO and CDC, the most prevalent symptom defining this syndrome (occurring in 60–70% of cases) is severe fatigue, significantly disrupting daily activities and akin to myalgia encephalomyelitis or chronic fatigue syndrome. Persistent shortness of breath, often accompanied by a cough and non-specific chest pain, is a hallmark manifestation of this disorder that lingers for an extended duration [3]. Neurocognitive symptoms include reduced concentration (brain fog), alterations in memory, headaches, and anosmia. Anxiety, depression, along with sleep disturbances, is also frequently reported. Additional manifestations encompass hair loss, joint pain, muscle aches, rapid heartbeat, and gastrointestinal rhythm disturbances. There have been descriptions of over 50 different symptoms, mostly ranging from mild to moderate, significantly impacting the affected individuals’ quality of life [6]. Reports from Iran indicate that between 64.2% and 79% of patients experienced at least one symptom in the post-acute stage, occurring 4–24 weeks after the onset of the disease, with nervousness and fatigue being the most commonly reported symptoms [7, 8].

Assessing and quantifying the dimensions of this syndrome plays a crucial role in categorizing the impacts and healthcare requirements of affected individuals. One of the noteworthy global scales for post-Covid morbidity assessment is the COVID-19 Yorkshire Rehabilitation Scale (C19‐YRS), developed and psychometrically evaluated in 2021 by O’Connor et al. [9]. This scale was specifically crafted to document the symptoms, functional performance, and disability experienced by patients’ post-Covid contraction. Its clinical utility and psychometric properties were thoroughly assessed in a prospective observational study involving 187 patients referred consecutively to a post-Covid complications clinic. The findings highlighted that the C19-YRS proved to be clinically effective and met the established psychometric criteria, offering initial evidence of its suitability as a tool to measure PCS [9].

The C19-YRS is a comprehensive 17-item self-reported assessment tool created to evaluate the enduring effects of COVID-19 on activities and participation, aligning with the International Classification of Functioning, Disability, and Health, as well as the Rehabilitation Impact Assessment in PCS [10]. This questionnaire comprises physician-completed, self-report, and digital versions. Currently, the C19-YRS is being implemented in the United Kingdom and utilized across 26 National Health Service (NHS) PCS services. It forms an integral part of the initial specialist PCS community rehabilitation services in the UK [9].

Research has demonstrated a higher susceptibility to COVID-19 and its associated complications among the elderly compared to other age demographics [11]. The long-term consequences of Covid-19, particularly among the elderly population, remain inadequately understood. Implementing tools capable of screening for Post-Covid Syndrome (PCS) and assessing its impact can facilitate tailored rehabilitation strategies following Covid-19, especially within this vulnerable age group. This investigation also offers significant insights pivotal for shaping upcoming research and clinical approaches aimed at addressing post-Covid morbidity among the elderly population. This study represents the first endeavor to evaluate the psychometric attributes of the C19-YRS within the realm of post-Covid morbidity assessment in elderly individuals.

## Method

### Study design and participant characteristics

The cross-sectional study was carried out in September 2022 within the comprehensive urban health service centers located in Babol, situated in northern Iran. The primary objective was to conduct a psychometric evaluation of the C19-YRS specifically focusing on the elderly population. The sample pool comprised 230 elderly individuals who had previously tested positive for Covid-19 via PCR testing. Inclusion criteria encompassed individuals aged between 60 and 84 years (Young and old seniors) [12], without severe cognitive impairments, lacking limitations in hearing, vision, or language, devoid of a history of terminal illness or severe acute ailment within the past 3 months, not undergoing medication for Alzheimer’s or depression. PCS is characterized by a set of persistent physical, cognitive, or psychological symptoms lasting from 12 weeks to a year post the disease’s onset, without attributable causes [13]. The variables to be assessed encompassed personal characteristics and the administration of the C19-YRS. Each participant completed an informed consent form. Approval for this study was granted by the ethics committee (IR.MUBABOL.HRI.REC.1401.134) at Babol University of Medical Sciences. Additionally, permission was obtained from the original authors who developed the English version of the assessment tool.

### Sampling method

The elderly individuals with Covid-19 were identified through the Babol University Health Vice-Chancellor’s Covid-19 portal system. A multi-stage sampling approach was employed to select the study participants. Babol city’s municipal districts served as the primary division, with two districts designated as separate classes. Two comprehensive health care centers were randomly chosen from each class. Subsequently, within each center, the required number of elderly individuals (proportional to their representation) was randomly selected from the eligible seniors’ list using a simple random method. Initially, potential participants were contacted via phone to explain the study’s purpose and procedures. Those who agreed and met the study criteria were invited to the designated health care centers on specified dates. Upon their arrival, participants completed written consent forms, following which the researcher conducted interviews and administered the necessary questionnaires. For psychometric studies, established references suggest a minimum of 10 individuals per question item [14]. Therefore, given the 17 questions in the utilized questionnaire, a minimum sample size of 170 participants was deemed necessary for this study.

### C19-YRS to measure Post-Covid-19 complications

The English version of the C19-YRS was developed and its psychometrics assessed by Sivan et al. [9] in 2022. In a prospective observational study involving 187 patients referred consecutively to a post-Covid complications clinic, the scale’s clinical utility and psychometric properties were evaluated. This scale was specifically designed to capture symptoms severity, functional capacity, and disability in patients’ post-Covid contraction. Psychometric findings indicated strong internal consistency (Cronbach’s α = 0.891), demonstrating robust reliability. Additionally, correlations between patients’ perceptions of overall health and their self-reported symptoms, function, and disability were notably aligned [9].

Comprising 17 questions and 4 subscales, the C19-YRS delineates symptom severity (questions 1–10), functional ability (questions 11–15), overall health (question no. 16), and 25 options for other symptoms. Notably, the other symptoms subscale, although providing supplementary clinical information, was not included in the assessment of symptom severity. The scoring system allocates 4 points to the symptom severity and functional ability subscales, ranging from 0 (indicating no symptoms) to 3 (representing the most severe symptoms). Consequently, these subscales have a potential score range of 0–30 and 0–15, respectively. The overall health subscale ranges from 0 to 10 points, while the other symptoms subscale ranges from 0 to 25 points. The total score is not calculated; each subscale is analyzed independently. To access and use this questionnaire, necessary communication and written consent were obtained via email from the primary researcher [9]. Moreover, the questionnaire is accessible freely with permission from the University of Leeds, England, using the link provided below: https://lensing.leeds.ac.uk/product/c19-yrs-covid-19-yorkshirerehabilitation-scale.

### Psychometric steps of the C19-YRS

#### Steps of translation and retranslation

The process used for translating the English version of the C19-YRS into Farsi followed a rigorous three-step method. Initially, two translators specialized in the health field translated the questionnaire from English to Farsi. Their focus was on ensuring meaningful translations that captured clarity, simplicity, brevity, and considerations of the audience, age group, and cultural aspects rather than literal word-for-word translations. Next, the Farsi version was re-translated back into English by two proficient translators, who were unaware of the questionnaire’s original content. This step aimed to validate the accuracy of the Farsi translation and ensure its fidelity to the original English version. In the final stage, a panel of experts convened in a meeting with the researchers to evaluate the quality of the translations. Any discrepancies or mismatches identified in the translations were discussed, and alternative wording or adjustments were proposed and incorporated to improve the accuracy and alignment of the Farsi translation with the original English version.

#### Face validity and content validity

The study ensured face validity of the questionnaire by involving 20 patients (comprising 10 men and 10 women) who had similar conditions as the target population. In addition, six experts evaluated the content validity of the instrument (two rehabilitation specialists, one instrument development expert, two specialists in infectious diseases, and one community health nursing specialists). The participants were asked to provide feedback on various aspects including clarity, readability, writing style, difficulty in understanding specific items, confusing words, comprehensibility, inadequacy, and ambiguity. Corrections were made in the Persian version based on the feedback received from this patient-expert group. Regarding content validity, this study did not conduct a separate assessment, as it was assumed that the questionnaire designers already took steps to establish content validity [15]. In instances where cultural inconsistencies arose in specific question items, resolution was achieved during the translation and retranslation phases of the study. This process ensured that cultural nuances were appropriately addressed in the Persian version of the questionnaire.

#### Reliability (internal consistency)

The study computed Cronbach’s alpha for both the overall scale and each individual subscale [16]. Values exceeding 0.7 were deemed satisfactory as per established standards [17]. Additionally, intraclass reliability was evaluated using a test-retest method. This involved 50 elderly patients who met the inclusion criteria and completed the questionnaire twice, with a two-week interval [18]. The obtained scores from these two stages were then analyzed using the intraclass correlation coefficient (ICC). Interpretation of the ICC values indicates that a range of 0.4–0.59 is acceptable, 0.6–0.74 is considered good, and values higher than 0.74 are deemed excellent for test-retest reliability [19].

#### Construct validity - exploratory factor analysis (EFA)

In this study, as a secondary analysis, the construct validity of the questionnaire among the Iranian population was examined to ascertain whether the scales derived from the questionnaire’s questions align with the initial dimensions or reveal new factors. Specifically, 16 questions related to three crucial scales of the questionnaire—symptom severity, functional ability, and overall health—were considered for this analysis. Exploratory Factor Analysis (EFA) using Principal Axis Factor analysis and Varimax Rotation was employed [20]. To evaluate sampling adequacy for factor analysis, the Kaiser-Meyer-Olkin (KMO) measure was calculated. A value exceeding 0.6 indicates that the data’s correlations are suitable for factor analysis. Additionally, the significance of Bartlett’s test was assessed to confirm the relationship between the variables (questionnaire items).

The total variance of each observed variable, formed with other variables in the factor, was estimated, with an Eigen value exceeding one considered as a criterion for introducing an additional factor in the model. Factor loadings ≥ 0.4 were deemed acceptable based on the factor analysis results [21]. Subsequently, the questions within each subscale were examined for their correlation with the total score of that particular subscale, utilizing the Pearson correlation coefficient. All analyses were conducted using SPSS and Amos software version 22, with a significance level set at 0.05.

#### Confirmatory factor analysis (CFA)

To evaluate and confirm the construct validity in an Iranian population, CFA was used based on the questions and scales of the primary standard questionnaire. Goodness of model fit indices was used to perform CFA. In addition to the Chi-square statistic and its significance level, three important indicators for the goodness of fit of the model in this study were: the chi-square index to the degree of freedom, the comparative fit index (CFI), and the root mean square error approximation index (RMSEA). The CFI index ranges from zero and one. Larger index values demonstrate better fit of the model. If this value is above 0.90, it is good. The lower the RMSEA values, the better the model fit, which should be less than 0.08 [22]. Other CFA indicators along with convergent and discriminate validity indices are listed in supplementary Tables 1 and 2 of the respectively.

## Results

The study participants had a mean age of 67.53 ± 5.72 years. Table 1 presents an overview of the participants’ characteristics.

**Table 1:** The participants’ characteristics by hospitalization due to covid-19

Variables		Total (*n* = 230)	Not hospitalized (*n* = 170)	Hospitalized (*n* = 60)
Gender	Female	(54.3)125	97(%57.1)	28 (%46.7)
	Male	(45.7)105	73(%42.9)	32(%53.3)
Education	Non -academic	(82.6)190	134(%78.8)	56(%93.3)
	Academic	(17.4)40	36(%21.2)	4(%6.7)
Marital status	Single	46(20.0)	34(%20.0)	12(%20.0)
	Married	(80.0)184	136(80.0)	48(80.0)
Occupation	At home	166(72.2)	124(72.9)	42(70.0)
	Outdoors	(27.8)64	46(27.1)	18(30.0)
Pension	No	(38.7)89	64(37.6)	25(41.7)
	Yes	(61.3)141	106(62.4)	35(58.3)
Coexistence	Single	31(13.5)	23(13.5)	8(13.3)
	With spouse/wife	115(50.0)	83(48.8)	32(53.3)
	With spouse/wife and child	70(30.4)	54(31.8)	16(26.7)
	With children	14(6.1)	10(5.9)	4(6.7)
Smoking/hookah/alcohol use	No	(94.8)218	159(93.5)	59(98.3)
	Yes	(5.2)12	11(6.5)	1(1.7)
Exercise	No	(35.7)82	61(35.9)	21(35.0)
	Yes	(64.3)148	109(64.1)	39(65.0)
Comorbidity	No	(17.0)39	35(20.6)	4(6.7)
	Yes	191(83.0)	135(79.4)	56(93.3)
Polypharmacy	No	(47)108	89(52.4)	19(31.7)
	Yes	122(53.0)	81(47.6)	41(68.3)
Health perception	No different/worse than others	62(27.0)	36(21.2)	26(43.3)
	Better than others	(73.0)168	134(78.8)	34(56.7)
BMI	Overweight/obese	146(63.5)	85(50.0)	24(40.0)
	Normal	(36.5)84	85(50.0)	36(60.0)

Values are frequency (percent)

Significant correlations, ranging from moderate to strong (*p* < 0.05), were observed among the questions within the C19-YRS subscales. Furthermore, an examination of the correlation between the questionnaire’s subscales revealed a strong and inverse relationship between symptom severity and overall health subscales. The most robust positive correlation was identified between symptom severity and functional ability. Statistically significant correlations were found among all subscales of the C19‐YRS (*p* < 0.05), indicating a coherent internal structure among the subscales (Table 2).

**Table 2:** C19-YRS subscales correlation

Subscales	Symptom severity	Functional ability	Other symptoms	Overall health
Symptom severity	1			
Functional ability	*r* = 0.696 p value = 0.001	1		
Other symptoms	*r* = 0.631 p value = 0.001	*r* = 0.610 p value = 0.001	1	
Overall health	*r* = − 0.795 p value = 0.001	*r* = − 0.702 p value = 0.001	*r* = − 0.642 p value = 0.001	1

Table 3 reports the reliability of C19-YRS subscales, presenting Cronbach’s alpha and intraclass correlation coefficient (ICC) at the 95% confidence interval. The ICC results for all subscales ranged from 0.98 to 1.00, exceeding 0.7 and indicating high reliability and precision of the standard questionnaire within this study’s population. The total Cronbach’s alpha for C19-YRS questions was 0.903, while for the subscales, it ranged from 0.730 to 0.890, which was deemed acceptable.

**Table 3:** Psychological properties of the C19-YRS

Subscales	Options number	Mean (standard deviation)	SE	SEM	Max - Min	Median (interquartile range)	Cronbach’ alpha	ICC	CI 95%
Symptom severity	10	6.32(5.00)	0.33	0.50	0–19	6(2–10)	0.890	0.998	0.997–0.999
Functional ability	5	1.75(2.20)	0.14	0.00	0–10	1(0–3)	0.877	1.00	1.00–1.00
Other symptoms	25	3.80(2.84)	0.18	0.13	0–14	3(2–6)	0.730	0.998	0.996–0.999
Overall health	1	7.36(1.26)	0.08	0.18	4–10	8(7–8)	–	0.980	0.964–0.988

SEM: standard error of measurement, ICC: intraclass correlation coefficient, CI: confidence interval

The assessment of the novel approach within Iranian society for establishing structural validity using EFA aims to explore whether existing subscales derived from the questions align or if new subscales emerge from this dataset. The KMO measure yielded a value of 0.903, and the Bartlett test resulted in significance (*p* < 0.001). These outcomes confirmed the adequacy of sampling and indicated that the data were suitable for factor analysis. EFA conducted on the 16 items of the C19-YRS revealed three factors, as indicated by Eigen values exceeding one and the Scree Plot, collectively explaining 57.46% of the total variance. These factors accounted for: the first factor at 23.94%, the second factor at 17.32%, and the third factor at 16.19% (Supplementary Fig. 1).

Based on factor loadings exceeding 0.4, the initial factor encompassed questions 7, 10, 9, 12, 5, 16, 3, and 8. Despite question 6 exhibiting a lower factor loading, it was included in this factor due to its significance in assessing the elderly. This newly established factor was labeled “Morbidity.” The second factor comprised questions 11, 13, 14, and 15, categorized under the new factor termed “Activities.” The third factor included questions 1, 2, and 4, forming a new factor termed “Body Functional Disorders” (Supplementary Table 3).

**Fig. 1:**
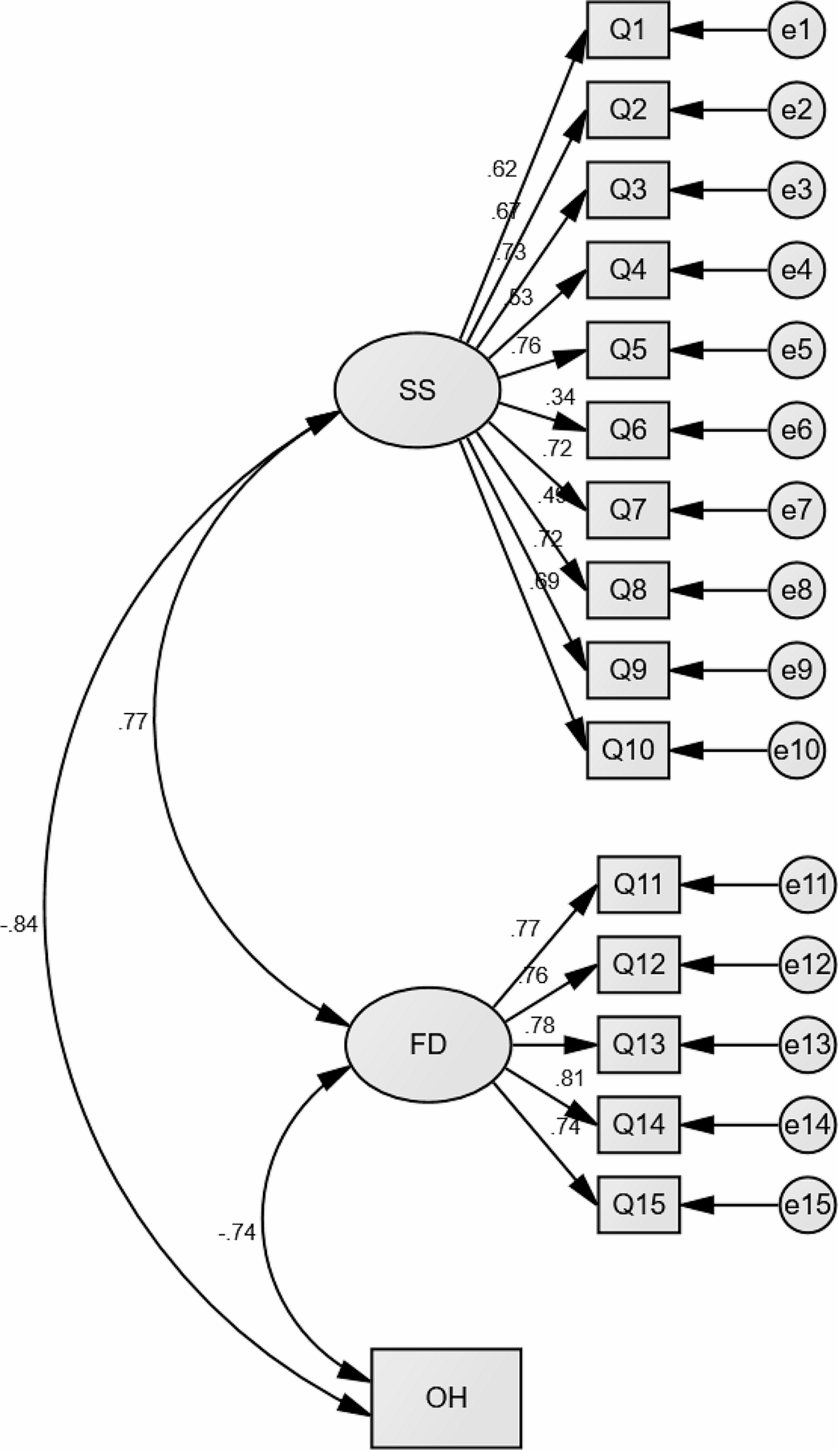
Confirmative factor analysis model for C19-YRS. SS: symptom severity, FD: functional disability, OH: overall health

The outcomes of the CFA applied to the standard questionnaire within this study’s population demonstrated standardized coefficients among the subscales as follows: symptoms severity and functional ability exhibited a coefficient of 0.77, symptoms severity correlated with overall health at −0.84, functional ability demonstrated a correlation with overall health at −0.74. These coefficients indicate strong correlations between the subscales within the model (Fig. 1). Also, model’s fit indices, including Chi-Square/Degrees of Freedom (χ2/df) at 4.89, indicated relatively acceptable values. However, the CFI at 0.811 and the RMSEA at approximately 0.130 were not within the optimal range for a good model fit, although the RMSEA approached a better fit.

## Discussion

The study aimed to validate the C19-YRS in Iranian elderly with PCS. The main scale comprises 17 items in 4 subscales. Notably, the ‘other symptoms’ subscale was excluded from the main analysis because its main purpose was to increase the health team’s understanding of COVID-19 symptoms. In O’Connor et al.‘s study [9], Cronbach’s alpha was reported as 0.89 for the total scale, 0.79 for symptom severity, and 0.79 for functional disability, compared to 0.93, 0.89, and 0.88, for the total scale and similar subscale in the present study.

The EFA conducted in this study served as an additional aspect in appraising the C19-YRS, owing to its novelty and absence of prior application in other research. It was run only in response to the question whether the scales defined based on these questions in the Iranian population will be the same as the initial questions or new dimensions will be identified. Although CFA was the main aim, conducted with 230 elderly individuals, the EFA results revealed a final model with 16 items and 3 factors, akin to the original model. In contrast to the original model, this study introduced the ‘morbidity’ factor by reassigning four items (1, 2, 4, and 6) to other subscales due to low factor loadings, while item 12 replaced them, resulting in an improved model fit. Notably, items 1, 2, and 4 were seldom reported as severe symptoms by participants, with fewer than 5% assigning a score of 3 to each. This indicates that these symptoms were particularly bothersome to a small subset of elderly individuals. Conversely, ‘walking or moving around’ (item 12) induced discomfort. Despite its lower factor loading, item 6 was retained in the first factor due to its significance among the elderly and its identification as a post-COVID-19 complication in certain studies [23, 24].

In this study, the second factor, labeled ‘activities,’ excluded item 12 (which had high saturation in the first factor), resulting in this factor consisting of four items (11, 13, 14, 15). A cohort study of Canadians over 50 years of age demonstrated that a COVID-19 diagnosis was significantly linked to deteriorated mobility and functional outcomes, even without hospitalization [25]. Their findings suggest the need for interventions aimed at individuals with mild to moderate COVID-19 not requiring hospitalization. Hence, movement disorder could be regarded as a symptom of severity or ‘morbidity’ associated with the post-COVID-19 syndrome.

Factor 3, denoting body functional disorders based on the semantics of the content within them encompasses three items (1, 2, 4). Broadly, this factor represents symptoms such as breathe shortness, cough/voice change, and smell and taste disturbance. It is believed that PCS to be a condition potentially impacting multiple organ systems. The intensified inflammatory response might involve various organs, leading to diverse manifestations [26]. For instance, SARS-CoV-2 infection in lung tissue triggers an inflammatory immune response that damages the airway’s epithelial cells, hindering proper gas exchange and resulting in fluid accumulation in the lungs [6].

Since the original model was standardized and previously presented in another study, this research focused solely on confirming the model’s applicability within an Iranian population. The results from CFA indicate that this model aligns closely with acceptable and ideal indicators in the Iranian context. Comparing the Cronbach’s alpha obtained in this study with the original version reveals relatively improved internal consistency values, except for question 6, which displayed a low factor loading due to participants’ potential difficulty in understanding the concept of cognitive impairment. The factor pertaining to symptom severity demonstrated the highest alpha value, suggesting its potential consideration in post-COVID-19 rehabilitation programs for the elderly. Strong correlations were observed among the three subscales—symptom severity, general health symptoms, and functional ability—signifying a consistent internal structure. As no similar articles for comparison exists, determining sensitivity and specificity of the scale was not feasible. Recently, the validity of the C19-YRS tool was evaluated in individuals over 20 years old in Thailand, among recovered COVID-19 patients. The analysis demonstrated acceptable validity and reliability of the Thai C19-YRS instrument for assessing psychometric variables within the Thai community. While 14 items displayed acceptable internal consistency based on corrected item correlation, five symptom severity items and two functional ability items were excluded. The final C19-YRS Cronbach’s alpha coefficient of 0.723 indicates satisfactory internal consistency and instrument reliability. However, further studies are warranted to standardize the diverse applications of this tool [27].

The C19-YRS represents the pioneering questionnaire designed to evaluate the enduring effects of COVID-19 on activities and participation within the framework of the International Classification of Functioning, Disability, and Health, as well as the PCS rehabilitation impact assessment [9]. Notably, structured questionnaires that specifically identify the long-term complications of COVID-19 are scarce. Sorensen et al. conducted a nationwide questionnaire study in Denmark [28] focusing on PCS, encompassing inquiries about recent symptoms, pre- and post-diagnosis health conditions, and self-reported physical and neurological symptoms within a specified timeframe. Another tool, the Symptom Burden Questionnaire for Long Covid (SBQ-LC), developed in England, evaluates a patient’s symptom burden over a week, consisting of 17 items and assessing the frequency and intensity of symptoms causing physiological and emotional responses [29]. Furthermore, Kayaaslan et al. introduced a specialized scale addressing persistent symptoms beyond 12 weeks from initial diagnosis. This scale, inspired by the C19-YRS tool, assesses characteristics of acute COVID-19, persistent symptoms categorized by systems, and information about outpatient clinic visits following recovery [30].

In the translation process, certain limitations of the study need to be considered. While acknowledging the potential for reliability and validity estimates from small sample sizes, this study recognizes its use of a relatively small sample. Furthermore, as the scale relies on self-reporting, its applicability to patients experiencing severe fatigue or communication impairments requires further investigation. Despite these limitations, the C19-YRS could be a useful adjunct to current post-COVID-19 assessments in clinical studies and used to complement clinician symptom assessment criteria. Additionally, the items in the scale provide clinicians with qualitative information to assist in targeting their clinical interventions to individual needs. This method has advantages over other methods because it may be used in any setting, does not require an external evaluator, and is not laboratory-based or requires special equipment. Most importantly, it measures the patients’ point of view. On the other hand in the present study, to accurately identify post-Covid-19 complications, only patients who had a definite PCR test were included in the study. Also, our study population was homogenous in terms of age and some health characteristics, and this error in predicting post-covid complications is reduced.

## Conclusion

Collecting data for research purposes requires appropriate tools and methodologies. In the context of this study, there was an initial validation of the items used to assess post-COVID complications among elderly Iranians, indicating their reliability and validity. However, during exploratory factor analysis within the Iranian population, new scales emerged from these questions. This indicates that the original questions might measure different aspects or categories of post-COVID complications beyond what was initially anticipated. To improve the questionnaire, further studies with larger sample sizes encompassing diverse populations are necessary. This broader research scope should include individuals from varied cultural, social, and economic backgrounds. Doing so will enhance the generalizability of findings, allowing for a more comprehensive understanding of post-COVID complications across different demographic groups.

### Electronic supplementary material

Below is the link to the electronic supplementary material.

**Supplementary Material 1:** Supplementary results

## Data Availability

The data supported during the present research are available from the corresponding author upon reasonable request.

## Abbreviations

C19-YRS: The COVID-19 Yorkshire Rehabilitation Scale
NICE: National Institute for Care Excellence
PCS: Post-Covid syndrome
CFA: Confirmatory Factor Analysis
RMSEA: Root mean square error approximation index
CFI: Comparative fit index
EFA: Exploratory Factor Analysis
KMO: Kaiser-Meyer-Olkin
