# Supplementary material for: Psychometric properties of the COVID-19 Yorkshire Rehabilitation Scale: Post-Covid-19 syndrome in Iranian elderly population
Source: BMC Infect Dis. 2024 Jan 11;24:77. doi: 10.1186/s12879-024-08991-0 (PMC10785474; doi:10.1186/s12879-024-08991-0)
Supplement: Supplementary file 1 — Supplementary Material 1: Supplementary results [file 12879_2024_8991_MOESM1_ESM.docx]

**Supplementary Table 1.** Fit model indices of the CFA of the Persian version of C19-YRS

| **Indices**  **model** | **χ2** | **df** | ***p***  **value** | **χ2/ df** | **RMSEA** | **PCLOSE** | **PCFI** | **AGFI** | **GFI** | **IFI** |
| --- | --- | --- | --- | --- | --- | --- | --- | --- | --- | --- |
| **CFA**  **model** | 498.92 | 102 | <0.001 | 4.89 | 0.130 | 0.850 | 0.689 | 0.777 | 0.811 | 0.812 |

χ2: chi-square, χ2/ df: chi-square/degree-of-freedom ratio, RMSEA: root mean square of error of approximation, PCLOSE: for close fit of the population RMSEA, PCFI: parsimonious comparative fit index, AGFI: adjusted goodness-of-fit index, GFI: goodness-of-fit index, IFI: incremental fit index

**Supplementary Table 2.** The indices of convergent and discriminate validity

| **Factors** | **CR** | **AVE** | **MSV** |
| --- | --- | --- | --- |
| **Symptom severity** | 0.374 | 0.415 | 0.387 |
| **Functional ability** | 0.739 | 0.539 | 0.158 |
| **Overall health** | - | - | - |

CR: Composite Reliability, AVE: Average Variance Extracted, MSV: Maximum Shared Squared Variance

**
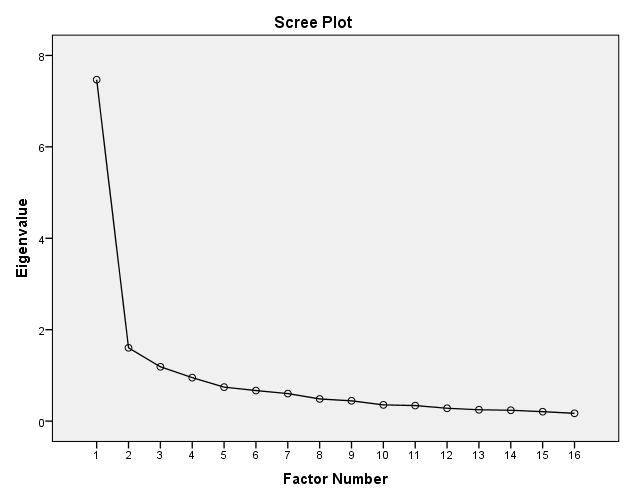
**

Supplementary **Figure 1.** Scree Plot in exploration factor analysis to identify the number of factor loads C19-YRS

**Supplementary Table 3.** Factor loads for C19-YRS questions in exploratory factor analysis using Varimax rotation in the elderly population with Covid-19 history

| **C19-YRS Items** | **Factor 1** | **Factor 2** | **Factor 3** |
| --- | --- | --- | --- |
| **Q7** | 0.776 | 0.191 | 0.162 |
| **Q10** | 0.692 | 0.165 | 0.236 |
| **Q9** | 0.692 | 0.302 | 0.183 |
| **Q12** | 0.661 | 0.401 | 0.261 |
| **Q5** | 0.602 | 0.191 | 0.400 |
| **Q16** | -0.538 | -0.432 | -0.493 |
| **Q3** | 0.527 | 0.202 | 0.459 |
| **Q8** | 0.441 | 0.142 | 0.204 |
| **Q6** | 0.252 | 0.171 | 0.144 |
| **Q13** | 0.157 | 0.865 | 0.163 |
| **Q15** | 0.221 | 0.806 | 0.078 |
| **Q14** | 0.435 | 0.600 | 0.211 |
| **Q11** | 0.474 | 0.569 | 0.200 |
| **Q2** | 0.220 | 0.169 | 0.849 |
| **Q1** | 0.198 | 0.174 | 0.755 |
| **Q4** | 0.264 | 0.052 | 0.565 |
| ***Eigen values*** | 3.83 | 2.77 | 2.59 |
| ***Variance explained*** | 23.94 | 17.32 | 16.19 |
| ***Cumulative variance*** | 57.46 | | |
